# Redetermination of the crystal structure of tetra­lithium octa­fluorido­zirconate(IV), Li_4_ZrF_8_, from single-crystal X-ray data

**DOI:** 10.1107/S2056989018018194

**Published:** 2019-01-04

**Authors:** Alexander T. Chemey, Thomas E. Albrecht-Schmitt

**Affiliations:** a Department of Chemistry and Biochemistry, Florida State University, 95 Chieftan Way, Tallahassee, FL 32306, USA

**Keywords:** crystal structure, redetermination, zirconium, metal fluoride, fluoride, ionic compounds

## Abstract

Presented herein is the crystal-structure redetermination of Li_4_ZrF_8_ from single-crystal X-ray data. Alkali zirconium fluorides are important in nuclear-relevant technologies, and zirconium is commonly employed as an analogue for tetra­valent *f*-block elements. The previous structure report of this species is based on powder X-ray data [Dugat *et al.* (1995 ▸). *J. Solid State Chem.* **120**, 187–196] but there has never been a refined structure model from single-crystal data. The octa­fluorido­zirconate moieties are held together by electrostatic attraction to lithium ions without sharing of fluoride sites between zirconium(IV) ions.

## Abstract 2

The structure of tetra­lithium octa­fluorido­zirconate has been redetermined by high-resolution single-crystal X-ray diffraction. This result is largely consistent with a prior report, but with significant improvements in precision.

## Chemical context   

Zirconium fluorides are commonly examined as members of trinary and ternary-phase alkali/transition metal/actinide fluorides for molten-salt reactors. Many of these molten salts incorporate lithium, because of the favorable nuclear and thermal properties of lithium fluoride. Compounds of zirconium are a useful (if imprecise) structural surrogate for tetra­valent cerium, thorium, uranium, and plutonium structures where these materials are unavailable or impractical (Thoma *et al.*, 1965 ▸, 1968 ▸). With the increased inter­est in carbon-neutral energy sources, investigations of nuclear-relevant technologies such as molten-salt reactors are of increasing inter­est. As a result, a re-evaluation of data is necessary in some areas. High-quality structure models of Li_2_ZrF_6_ and Li_3_Zr_4_F_19_ from single-crystal data have previously been discussed in the literature (Brunton, 1973 ▸; Dugat *et al.*, 1995 ▸). The structure of Li_4_ZrF_8_ was reported to be isotypic to the uranium species by powder X-ray diffraction (Dugat *et al.*, 1995 ▸), but no refined structure model from single-crystal data has been reported to date.

## Structural commentary   

**Figure 1:**
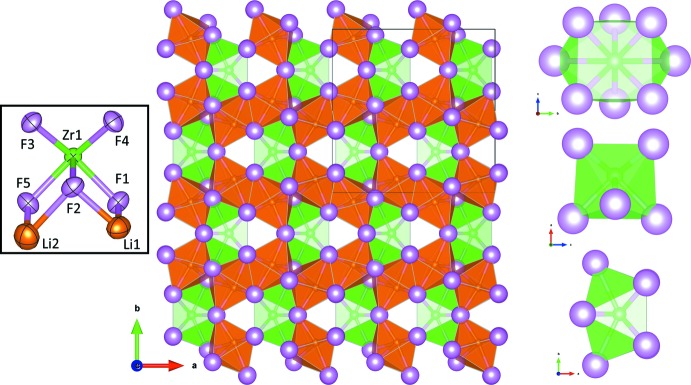
The crystal structure of Li_4_ZrF_8_. The large image on the left is of the crystal packing down the *c* axis. The inset demonstrates the displacement ellipsoids of all ions at the 95% probability level. From top-to-bottom on the right, the views of the ZrF_8_^4−^ unit down the *a*, *b*, and *c* axes. Color code: green, zirconium; orange, lithium; pink, fluorine.

Li_4_ZrF_8_ is confirmed to be isotypic with the reported structures of Li_4_ *M*F_8_ (*M* = Tb, U) (El-Ghozzi *et al.*, 1992 ▸; Brunton, 1967 ▸). The zirconium(IV) ion is surrounded by eight fluoride ions in a bicapped trigonal prism (Fig. 1 ▸), while both of the two unique lithium sites are surrounded by six fluoride ions in slightly distorted octa­hedra. Zr—F bond lengths range from 2.0265 (9) to 2.2550 (7) Å (Table 1 ▸), and Li—F bonds range from 1.931 (3) to 2.204 (3) Å. The octa­fluorido­zirconate anion is isolated, separated by 4.9906 (4) Å from its crystallographic nearest neighbors. Investigation of several distinct crystals of different size and apparent crystal habit all resulted in unit-cell parameters that agreed with the published unit cell of Li_4_ZrF_8_. It is therefore likely that, despite the sub-stoichiometric ratio in the reaction (which was intended to produce other lithium zirconium fluorides), Li_4_ZrF_8_ is the most stable single-crystalline zirconate formed.

The refined crystal structure model is qualitatively very similar in most respects to that reported by Dugat *et al.* (1995 ▸), including the connectivity and zirconium bonding environment. There are significant statistical improvements in all major metrics, including unit-cell precision, standard uncertainties of the unit cell and bond lengths, and a much finer identification of the lithium and fluoride ion sites. Despite this concordance, every zirconium-fluoride bond length reported in the literature is more than one standard uncertainty apart from the zirconium–fluoride distances determined in the structure reported here. This is not a result of systematic bias in the calculated powder-pattern bond lengths. The eight Zr—F bonds are evenly split, with four longer than reported here, and four shorter, and the obtained average bond length is very close to the one from the previous study. Among the twelve lithium–fluoride bonds, the average bond lengths for each lithium site are statistically identical to those noted in the previous model, but distinct at the standard uncertainty in the data reported here. The site designated Li1 in each structure has greater asymmetry than its neighbor, but the re-examined data do not have a difference that is nearly so marked; the literature Li1—F bond lengths range from 1.84 (2)–2.11 (2) Å, while the new result reported here has bond lengths of 1.942 (2)–2.054 (2) Å. Additionally, the axes of the unit cell are different by a margin greater than the standard uncertainty reported in the literature, as all three axes reported here are greater in size. The overall effect on the unit-cell volume is small, however, but there is an additional order of magnitude of precision obtained. For more details, direct comparisons of the bond lengths and the unit cells are given in Table 1 ▸.

The crystal examined exhibited static disorder, observable by the zirconium site (which has significantly more electron density than the other atoms). Both zirconium sites are on Wyckoff position 4*c* (site symmetry. *m*.).

## Synthesis and crystallization   

Lithium fluoride (43.0 mg, 1.66 mmol; 99.85% Alfa Aesar) and zirconium dioxide (61.1 mg, 0.496 mmol; 99% Aldrich) were charged into an 8 mL PTFE-lined autoclave. 1.00 mL of deionized water was then added, followed by the dropwise addition of 1.00 mL 48% hydro­fluoric acid (Sigma–Aldrich). The autoclave was sealed, and heated at 473 K for twenty-four h, followed by controlled cooling to room temperature at a rate of 5 K h^−1^. The title product was isolated from the supernatant by repeatedly rinsing with chilled deionized water to dilute the fluoride hazard and to remove any lithium fluoride that remained in the HF solution. Methanol was used to transfer the samples to a petri dish, followed by drying in air. Large (up to 5 mm) crystals were parallelogram columns that cleaved into parallelepipeds, while small (50 µm-scale) crystals were thin parallelogram plates.

*Caution! Fluoride salts and hydro­fluoric acid are acute chemical haza­rds. Work was conducted in a well-ventilated fume hood, separate from other reactions. This reaction was conducted by a chemist experienced in metal-fluoride synthesis. Thick rubber gloves were worn over standard lab attire, as well as a rubber smock, and a plastic face shield.*

## Refinement   

Crystal data, data collection and structure refinement details are summarized in Table 2 ▸. *PLATON* (Spek, 2009 ▸) was used to check for unresolved solvent electron density, additional symmetry, or twinning. There was static disorder present in all crystals examined, and a high remaining electron-density peak assignable to a second Zr site (Zr2) was observed at approximately one-half of the *c* axis apart from Zr1. This disorder was resolved by the PART command (Sheldrick, 2015*b* ▸) with an occupancy ratio of 0.9611 (13):0.0389 (13) for Zr1:Zr2, and no other atoms were observed in the disordered second part. The minor disorder part is excluded from the illustrations and the bond-length analysis and comparison with the previous report.

## Supplementary Material

Crystal structure: contains datablock(s) I. DOI: 10.1107/S2056989018018194/wm5477sup1.cif

Structure factors: contains datablock(s) I. DOI: 10.1107/S2056989018018194/wm5477Isup2.hkl

CCDC reference: 1886828

Additional supporting information:  crystallographic information; 3D view; checkCIF report

## Figures and Tables

**Table 1:** Comparison of unit-cell parameters and bond lengths (Å) of Li_4_ZrF_8_ to those from the previous report

	**1995 study** ^*a*^	**This work**
*a*	9.581 (1)	9.5959 (3)
*b*	9.611 (1)	9.6218 (3)
*c*	5.663 (1)	5.6735 (2)
*V* (Å^3^)	521.47	523.83 (3)
Zr1—F1	2.06 (2)	2.0265 (9)
Zr1—F5	2.06 (2)	2.0419 (9)
Zr1—F3 (2×)	2.06 (1)	2.1020 (6)
Zr1—F4 (2×)	2.07 (1)	2.1109 (6)
Zr1—F2 (2×)	2.27 (1)	2.2550 (7)
Zr1—F (averaged)	2.12	2.124
Li1—F (averaged)	2.00	2.001
Li2—F (averaged)	2.06	2.059

(*a*) Dugat *et al.* (1995 ▸); atom labeling adapted to the current study.

**Table 2:** Experimental details

Crystal data	
Chemical formula	Li_4_ZrF_8_
*M*_r_	270.98
Crystal system, space group	Orthorhombic, *P* *n* *m* *a*
Temperature (K)	296
*a*, *b*, *c* (Å)	9.5959 (3), 9.6218 (3), 5.6735 (2)
*V* (Å^3^)	523.83 (3)
*Z*	4
Radiation type	Mo *K*α
μ (mm^−1^)	2.21
Crystal size (mm)	0.08 × 0.08 × 0.08

Data collection	
Diffractometer	Bruker D8 Quest
Absorption correction	Multi-scan (*SADABS*; Bruker, 2015 ▸)
*T*_min_, *T*_max_	0.067, 0.135
No. of measured, independent and observed [*I* > 2σ(*I*)] reflections	60244, 2776, 2299
*R*_int_	0.035
(sin θ/λ)_max_ (Å^−1^)	1.070

Refinement	
*R*[*F* ^2^ > 2σ(*F* ^2^)], *wR*(*F* ^2^), *S*	0.029, 0.073, 1.11
No. of reflections	2776
No. of parameters	71
Δρ_max_, Δρ_min_ (e Å^−3^)	1.67, −0.76

Computer programs: *APEX3* and *SAINT* (Bruker, 2015 ▸), *SHELXS2014* (Sheldrick, 2015*a* ▸), *SHELXL2014* (Sheldrick, 2015*b* ▸), *SHELXP2014* (Sheldrick, 2008 ▸), *VESTA* (Momma & Izumi, 2011 ▸) and *publCIF* (Westrip, 2010 ▸).

## supplementary crystallographic information

### Crystal data

**Table:**

Li_4_ZrF_8_	*D*_x_ = 3.436 Mg m^−^^3^
*M**_r_* = 270.98	Mo *K*α radiation, λ = 0.71073 Å
Orthorhombic, *P**n**m**a*	Cell parameters from 2777 reflections
*a* = 9.5959 (3) Å	θ = 4.2–49.5°
*b* = 9.6218 (3) Å	µ = 2.21 mm^−^^1^
*c* = 5.6735 (2) Å	*T* = 296 K
*V* = 523.83 (3) Å^3^	Parallelepiped, colorless
*Z* = 4	0.08 × 0.08 × 0.08 mm
*F*(000) = 496	

### Data collection

**Table:**

Bruker D8 Quest diffractometer	2299 reflections with *I* > 2σ(*I*)
Radiation source: Iµs microfocused	*R*_int_ = 0.035
0.5° wide /w exposures scans	θ_max_ = 49.5°, θ_min_ = 4.2°
Absorption correction: multi-scan (*SADABS*; Bruker, 2015)	*h* = −20→20
*T*_min_ = 0.067, *T*_max_ = 0.135	*k* = −20→20
60244 measured reflections	*l* = −12→12
2776 independent reflections	

### Refinement

**Table:**

Refinement on *F*^2^	71 parameters
Least-squares matrix: full	0 restraints
*R*[*F*^2^ > 2σ(*F*^2^)] = 0.029	*w* = 1/[σ^2^(*F*_o_^2^) + (0.0375*P*)^2^ + 0.2784*P*] where *P* = (*F*_o_^2^ + 2*F*_c_^2^)/3
*wR*(*F*^2^) = 0.073	(Δ/σ)_max_ < 0.001
*S* = 1.11	Δρ_max_ = 1.67 e Å^−^^3^
2776 reflections	Δρ_min_ = −0.76 e Å^−^^3^

### Special details

**Table:**

Geometry. All esds (except the esd in the dihedral angle between two l.s. planes) are estimated using the full covariance matrix. The cell esds are taken into account individually in the estimation of esds in distances, angles and torsion angles; correlations between esds in cell parameters are only used when they are defined by crystal symmetry. An approximate (isotropic) treatment of cell esds is used for estimating esds involving l.s. planes.

### Fractional atomic coordinates and isotropic or equivalent isotropic displacement parameters (Å^2^)

**Table:**

	*x*	*y*	*z*	*U*_iso_*/*U*_eq_	Occ. (<1)
Zr1	0.13558 (2)	0.2500	0.37104 (2)	0.00881 (3)	0.9611 (13)
Zr2	0.1348 (3)	0.2500	−0.1186 (6)	0.0116 (8)	0.0389 (13)
F1	0.28621 (10)	0.2500	0.12069 (15)	0.01443 (13)	
F2	0.23547 (7)	0.46213 (7)	0.37298 (11)	0.01531 (10)	
F3	0.02327 (7)	0.37844 (7)	0.60287 (11)	0.01393 (10)	
F4	0.02100 (7)	0.38245 (7)	0.14647 (11)	0.01463 (10)	
F5	0.28873 (10)	0.2500	0.62088 (16)	0.01499 (14)	
Li1	0.3693 (2)	0.4418 (3)	0.1187 (4)	0.0190 (4)	
Li2	0.3976 (3)	0.4187 (3)	0.6293 (4)	0.0228 (4)	

### Atomic displacement parameters (Å^2^)

**Table:**

	*U*^11^	*U*^22^	*U*^33^	*U*^12^	*U*^13^	*U*^23^
Zr1	0.00928 (5)	0.00787 (5)	0.00927 (5)	0.000	0.00044 (3)	0.000
Zr2	0.0113 (13)	0.0141 (14)	0.0094 (12)	0.000	0.0003 (9)	0.000
F1	0.0155 (3)	0.0129 (3)	0.0149 (3)	0.000	0.0043 (3)	0.000
F2	0.0154 (2)	0.0136 (2)	0.0170 (2)	−0.00122 (18)	0.00293 (19)	−0.00140 (17)
F3	0.0148 (2)	0.0117 (2)	0.0153 (2)	0.00101 (16)	0.00267 (17)	−0.00149 (17)
F4	0.0156 (2)	0.0134 (2)	0.0149 (2)	0.00135 (18)	−0.00247 (17)	0.00120 (17)
F5	0.0147 (3)	0.0152 (3)	0.0151 (3)	0.000	−0.0034 (3)	0.000
Li1	0.0180 (9)	0.0197 (10)	0.0194 (10)	0.0007 (7)	0.0018 (7)	0.0031 (7)
Li2	0.0254 (11)	0.0231 (11)	0.0200 (10)	−0.0057 (10)	−0.0013 (8)	−0.0019 (8)

### Geometric parameters (Å, º)

**Table:**

Zr1—F1	2.0265 (9)	F3—Li2^ix^	1.978 (3)
Zr1—F5	2.0419 (9)	F3—Li1^viii^	2.016 (3)
Zr1—F3	2.1020 (6)	F3—Li1^iii^	2.033 (2)
Zr1—F3^i^	2.1021 (6)	F3—Zr2^x^	2.274 (3)
Zr1—F4	2.1109 (6)	F4—Li2^iii^	1.993 (3)
Zr1—F4^i^	2.1109 (6)	F4—Li1^iii^	2.054 (2)
Zr1—F2^i^	2.2550 (7)	F4—Li2^vii^	2.069 (3)
Zr1—F2	2.2550 (7)	F5—Li2	1.931 (3)
Zr1—Li1^ii^	3.152 (3)	F5—Li2^i^	1.931 (3)
Zr1—Li1^iii^	3.152 (3)	F5—Zr2^x^	2.090 (3)
Zr1—Li1^i^	3.238 (2)	Li1—F2^vii^	1.952 (3)
Zr1—Li1	3.238 (2)	Li1—F3^vii^	2.016 (3)
Zr2—F1	1.988 (3)	Li1—F3^xi^	2.033 (2)
Zr2—F5^iv^	2.090 (3)	Li1—F4^xi^	2.054 (2)
Zr2—F4^i^	2.253 (3)	Li1—Li2^iv^	2.798 (4)
Zr2—F4	2.253 (3)	Li1—Li2^vii^	2.893 (4)
Zr2—F3^v^	2.274 (3)	Li1—Li2	2.918 (4)
Zr2—F3^iv^	2.274 (3)	Li1—Li2^xii^	2.974 (4)
Zr2—Li2^ii^	2.796 (4)	Li1—Li1^xiii^	3.059 (5)
Zr2—Li2^iii^	2.796 (4)	Li1—Zr1^xi^	3.152 (3)
Zr2—Li1^i^	3.206 (4)	Li2—F3^xiv^	1.978 (3)
Zr2—Li1	3.206 (4)	Li2—F4^xi^	1.993 (3)
Zr2—Li1^vi^	3.320 (3)	Li2—F4^viii^	2.069 (3)
Zr2—Li1^vii^	3.320 (3)	Li2—F2^viii^	2.204 (3)
F1—Li1^i^	2.010 (3)	Li2—Zr2^xi^	2.796 (4)
F1—Li1	2.010 (3)	Li2—Li1^x^	2.798 (4)
F2—Li1	1.942 (2)	Li2—Li1^viii^	2.893 (4)
F2—Li1^viii^	1.952 (3)	Li2—Li2^xii^	2.909 (6)
F2—Li2	2.170 (3)	Li2—Li1^xii^	2.974 (4)
F2—Li2^vii^	2.204 (3)		
			
F1—Zr1—F5	88.46 (4)	Li1^viii^—F2—Zr1	102.32 (9)
F1—Zr1—F3	143.536 (19)	Li2—F2—Zr1	97.69 (8)
F5—Zr1—F3	86.25 (3)	Li2^vii^—F2—Zr1	102.80 (8)
F1—Zr1—F3^i^	143.536 (19)	Li2^ix^—F3—Li1^viii^	96.28 (11)
F5—Zr1—F3^i^	86.25 (3)	Li2^ix^—F3—Li1^iii^	88.47 (12)
F3—Zr1—F3^i^	72.02 (4)	Li1^viii^—F3—Li1^iii^	98.15 (9)
F1—Zr1—F4	87.05 (3)	Li2^ix^—F3—Zr1	155.27 (10)
F5—Zr1—F4	142.549 (19)	Li1^viii^—F3—Zr1	105.69 (7)
F3—Zr1—F4	75.86 (3)	Li1^iii^—F3—Zr1	99.31 (8)
F3^i^—Zr1—F4	117.75 (3)	Li2^ix^—F3—Zr2^x^	81.93 (12)
F1—Zr1—F4^i^	87.05 (3)	Li1^viii^—F3—Zr2^x^	101.24 (9)
F5—Zr1—F4^i^	142.549 (19)	Li1^iii^—F3—Zr2^x^	159.16 (11)
F3—Zr1—F4^i^	117.75 (3)	Li2^iii^—F4—Li1^iii^	92.28 (11)
F3^i^—Zr1—F4^i^	75.86 (3)	Li2^iii^—F4—Li2^vii^	91.46 (11)
F4—Zr1—F4^i^	74.27 (4)	Li1^iii^—F4—Li2^vii^	92.35 (12)
F1—Zr1—F2^i^	72.556 (19)	Li2^iii^—F4—Zr1	152.74 (10)
F5—Zr1—F2^i^	71.98 (2)	Li1^iii^—F4—Zr1	98.35 (8)
F3—Zr1—F2^i^	138.33 (2)	Li2^vii^—F4—Zr1	112.96 (9)
F3^i^—Zr1—F2^i^	71.50 (2)	Li2^iii^—F4—Zr2	82.12 (12)
F4—Zr1—F2^i^	140.42 (2)	Li1^iii^—F4—Zr2	158.97 (11)
F4^i^—Zr1—F2^i^	71.21 (3)	Li2^vii^—F4—Zr2	107.98 (10)
F1—Zr1—F2	72.556 (19)	Li2—F5—Li2^i^	114.4 (2)
F5—Zr1—F2	71.98 (2)	Li2—F5—Zr1	114.00 (9)
F3—Zr1—F2	71.50 (2)	Li2^i^—F5—Zr1	114.00 (9)
F3^i^—Zr1—F2	138.33 (2)	Li2—F5—Zr2^x^	111.40 (10)
F4—Zr1—F2	71.21 (3)	Li2^i^—F5—Zr2^x^	111.40 (10)
F4^i^—Zr1—F2	140.42 (2)	F2—Li1—F2^vii^	98.16 (11)
F2^i^—Zr1—F2	129.68 (4)	F2—Li1—F1	79.97 (10)
F1—Zr2—F5^iv^	88.08 (13)	F2^vii^—Li1—F1	103.56 (12)
F1—Zr2—F4^i^	84.17 (11)	F2—Li1—F3^vii^	106.53 (14)
F5^iv^—Zr2—F4^i^	144.55 (6)	F2^vii^—Li1—F3^vii^	79.92 (11)
F1—Zr2—F4	84.17 (11)	F1—Li1—F3^vii^	172.28 (14)
F5^iv^—Zr2—F4	144.55 (6)	F2—Li1—F3^xi^	166.02 (16)
F4^i^—Zr2—F4	68.88 (10)	F2^vii^—Li1—F3^xi^	94.29 (10)
F1—Zr2—F3^v^	144.93 (7)	F1—Li1—F3^xi^	90.96 (12)
F5^iv^—Zr2—F3^v^	80.86 (10)	F3^vii^—Li1—F3^xi^	81.85 (9)
F4^i^—Zr2—F3^v^	85.89 (8)	F2—Li1—F4^xi^	90.85 (10)
F4—Zr2—F3^v^	122.89 (15)	F2^vii^—Li1—F4^xi^	163.74 (15)
F1—Zr2—F3^iv^	144.93 (7)	F1—Li1—F4^xi^	91.29 (12)
F5^iv^—Zr2—F3^iv^	80.86 (10)	F3^vii^—Li1—F4^xi^	84.55 (10)
F4^i^—Zr2—F3^iv^	122.89 (15)	F3^xi^—Li1—F4^xi^	78.64 (9)
F4—Zr2—F3^iv^	85.89 (8)	F5—Li2—F3^xiv^	100.62 (14)
F3^v^—Zr2—F3^iv^	65.85 (9)	F5—Li2—F4^xi^	98.91 (13)
F1—Zr2—Li2^ii^	127.56 (12)	F3^xiv^—Li2—F4^xi^	101.93 (15)
Zr2—F1—Li1^i^	106.62 (8)	F5—Li2—F4^viii^	169.37 (19)
Zr2—F1—Li1	106.62 (8)	F3^xiv^—Li2—F4^viii^	85.12 (11)
Li1^i^—F1—Li1	133.23 (14)	F4^xi^—Li2—F4^viii^	88.54 (11)
Li1^i^—F1—Zr1	106.68 (7)	F5—Li2—F2	75.95 (10)
Li1—F1—Zr1	106.68 (7)	F3^xiv^—Li2—F2	171.67 (16)
Li1—F2—Li1^viii^	156.83 (4)	F4^xi^—Li2—F2	86.18 (9)
Li1—F2—Li2	90.24 (11)	F4^viii^—Li2—F2	97.12 (13)
Li1^viii^—F2—Li2	88.97 (10)	F5—Li2—F2^viii^	98.01 (13)
Li1—F2—Li2^vii^	88.27 (10)	F3^xiv^—Li2—F2^viii^	88.47 (9)
Li1^viii^—F2—Li2^vii^	84.42 (11)	F4^xi^—Li2—F2^viii^	158.10 (16)
Li2—F2—Li2^vii^	159.38 (5)	F4^viii^—Li2—F2^viii^	73.03 (10)
Li1—F2—Zr1	100.73 (9)	F2—Li2—F2^viii^	84.53 (11)

Symmetry codes: (i) *x*, −*y*+1/2, *z*; (ii) *x*−1/2, −*y*+1/2, −*z*+1/2; (iii) *x*−1/2, *y*, −*z*+1/2; (iv) *x*, *y*, *z*−1; (v) *x*, −*y*+1/2, *z*−1; (vi) −*x*+1/2, *y*−1/2, *z*−1/2; (vii) −*x*+1/2, −*y*+1, *z*−1/2; (viii) −*x*+1/2, −*y*+1, *z*+1/2; (ix) *x*−1/2, *y*, −*z*+3/2; (x) *x*, *y*, *z*+1; (xi) *x*+1/2, *y*, −*z*+1/2; (xii) −*x*+1, −*y*+1, −*z*+1; (xiii) −*x*+1, −*y*+1, −*z*; (xiv) *x*+1/2, *y*, −*z*+3/2.
